# Complete nontuberculous mycobacteria whole genomes using an optimized DNA extraction protocol for long-read sequencing

**DOI:** 10.1186/s12864-019-6134-y

**Published:** 2019-10-30

**Authors:** Jennifer M. Bouso, Paul J. Planet

**Affiliations:** 1 Division of Pulmonary Medicine, Children’s Hospital of Philadelphia, Philadelphia, PA USA; 2 Division of Infectious Diseases, Children’s Hospital of Philadelphia, Philadelphia, PA USA; 3 Perelman School of Medicine, University of Pennsylvania, Philadelphia, PA USA; 4 Sackler Institute for Comparative Genomics, American Museum of Natural History, New York, NY USA

**Keywords:** Mycobacteria, Long-read sequencing, Whole genome sequencing, Nontuberculous mycobacteria, *Mycobacterium avium* complex, Mycobacterium abscessus complex, Genome assembly, Cystic fibrosis, Chronic obstructive pulmonary disease, Bronchiectasis

## Abstract

**Background:**

Nontuberculous mycobacteria (NTM) are a major cause of pulmonary and systemic disease in at-risk populations. Gaps in knowledge about transmission patterns, evolution, and pathogenicity during infection have prompted a recent surge in genomic NTM research. Increased availability and affordability of whole genome sequencing (WGS) techniques provide new opportunities to sequence and construct complete bacterial genomes faster and at a lower cost. However, extracting large quantities of pure genomic DNA is particularly challenging with NTM due to its slow growth and recalcitrant cell wall. Here we report a DNA extraction protocol that is optimized for long-read WGS of NTM, yielding large quantities of highly pure DNA with no additional clean-up steps.

**Results:**

Our DNA extraction method was compared to 6 other methods with variations in timing of mechanical disruption and enzymatic digestion of the cell wall, quantity of matrix material, and reagents used in extraction and precipitation. We tested our optimized method on 38 clinical isolates from the *M. avium* and *M. abscessus* complexes, which yielded optimal quality and quantity measurements for Oxford Nanopore Technologies sequencing. We also present the efficient completion of circularized *M. avium* subspecies *hominissuis* genomes using our extraction technique and the long-read sequencing MinION platform, including the identification of a novel plasmid.

**Conclusions:**

Our optimized extraction protocol and assembly pipeline was both sufficient and efficient for genome closure. We expect that our finely-tuned extraction method will prove to be a valuable tool in long-read sequencing and completion of mycobacterial genomes going forward. Utilization of comprehensive, long-read based approaches will advance the understanding evolution and pathogenicity of NTM infections.

## Background

The emergence of nontuberculous mycobacteria (NTM) infection in immunocompromised hosts, the elderly, patients with cystic fibrosis (CF), and patients with non-CF chronic lung disease (COPD, asthma, non-CF bronchiectasis) has prompted genomic investigations aimed at uncovering the determinants of pathogenicity, transmission, evolution, and adaptation [1–10]. Bacterial evolution and phylogenomic research have been revolutionized by more available and affordable whole genome sequencing (WGS) [11–15]. WGS of NTM has begun to shed light on taxonomic conundrums, transmissibility, and global evolution [16–24]. However, the unique challenges of slow growth rates and inefficient DNA extraction have impeded rigorous genomic investigation of NTM.

Over recent years, the vast majority of genomic analyses have relied on short-read, shot-gun sequencing (75–500 base pairs), which can deliver exceptional accuracy, but rarely produce closed genomes. Indeed, less than 10% of available microbial genomes are complete [25]. During comparative analyses, fragmented assemblies are problematic because they may unlink gene clusters, fail to resolve repetitive and G + C rich regions, neglect insertion and deletion elements (indels), and overlook recombination [26–29].

Long-read sequencing promises an enhanced ability to complete bacterial genomes. The most commonly available techniques for long-read sequencing are the Single Molecule Real-Time (SMRT) technology by Pacific Biosciences® (PacBio, United States) and the newer Oxford Nanopore Technologies (ONT, United Kingdom) MinION [14, 27, 30]. Unlike most short-read sequencing methods, which require only very small amounts of DNA (as low as 1 ng), long-read platforms require high quantities of very pure DNA for acceptable processing. DNA purity and integrity (i.e., length or molecular weight [MW]) is not only essential for functionality of the sequencer, but also is directly related to the quality of downstream bioinformatic analyses, as the DNA MW places a natural upper bound on the potential read length. ONT MinION sequencing requires input of 400–1000 ng of high MW DNA (average fragment size of > 30 kb) with low solvent/salt and protein contamination (optical density [OD] 260/230 2.0–2.2 and 260/280 ~ 1.8, respectively) (nanoporetech.com).

Extracting large quantities of intact, pure genomic DNA is exceptionally challenging with NTM due to their hardy, lipid-laden mycobacterial cell wall. Standard extraction techniques (i.e., commercial kits) do not yield sufficient quantities of DNA for WGS while overly vigorous techniques shear DNA into suboptimal MWs for long-read sequencing. In our experience, the standard protocols specific for mycobacterial DNA extraction were unable to yield DNA that was of sufficient quality for ONT MinION sequencing [31–36]. We thus developed an optimized protocol over the course of performing hundreds of NTM DNA extractions using components of several extraction techniques, initially through trial and error, and subsequently confirmed by direct comparisons as described here. Our optimized method varies from the widely-used Käser et al. method by early bead-beating (prior to enzymatic digestion, as opposed to after) in high concentrations of sodium dodecyl sulfate (SDS) followed by gentle gel-based extraction to protect long strands of DNA, and an isopropanol precipitation favoring DNA purification above DNA quantity.

The goal of developing our protocol was to extract large amounts of high MW, pure DNA for use in long-read WGS. To evaluate the subtle alterations in methodology, we compared 6 variations in design, demonstrate the superiority of our optimized technique, validate its use on a large number of clinical isolates of two NTM species complexes, and prove its capacity for producing sufficient reads by the ONT MinION sequencer for genome completion. We also present three complete and circularized genomes constructed with ONT reads as well as a novel plasmid.

## Results

### Method comparison, validation, sequencing, and assembly

#### Methods defined

Our full DNA extraction protocol can be found in Additional file 1. An experiment was designed to test 6 variables in a standard phenol-based extraction technique; variable choices were made based on a number of grid experiments completed previously (Additional file 2: Table S1). Alterations in methodology included the timing of mechanical disruption, the quantity of beads used for mechanical disruption, extraction with phenol versus chloroform-isoamyl alcohol only, precipitation with either cold ethanol or room temperature isopropanol, sodium chloride versus sodium acetate precipitation, and the number of final washes. Variations in methods are outlined in Table 1. Notably, all methods were performed in a “Total Lysis Buffer” (TLB) that was previously found to be superior in direct comparison to a standard buffer (Additional file 2: Table S1); see Additional file 1 for TLB composition. Method 3 is most similar to a standard protocol by Käser et al. [32]. Method 5 is our optimized method and was the only method to produce sufficient quantity and quality standards for the ONT MinION long-read sequencer.

**Table 1:** Differentiation of tested methods by variable

	Method 1	Method 2	Method 3	Method 4	Method 5	Method 6	Method 7
Early vs. Late bead-beating^*a*^	Early	Late	Late	Early	Early	Early	Early
Bead Quantity	150 mg	150 mg	150 mg	75 mg	150 mg	150 mg	150 mg
Phenol vs. No Phenol^b^	No phenol	No phenol	Phenol	No phenol	Phenol	No phenol	No phenol
Precipitation Temp/Reagent^c^	RT/2-Prop	RT/2-Prop	Cold ETOH	RT/2-Prop	RT/2-Prop	Cold ETOH	RT/2-Prop
Precipitation Salt^d^	NaOAc	NaOAc	NaOAc	NaOAc	NaCl	NaOAc	NaOAc
Number of washes	3	3	3	3	3	3	5

^a^ “Early” bead-beating refers to the timing prior *to* enzymatic digestion; “Late” bead-beating refers to timing after enzymatic digestion

All Early bead-beating was done in high SDS concentration, see Additional file 1

^b^ DNA extractions in “no phenol” were extracted as described in Methods with chloroform:isoamyl alcohol (24:1, Tris-saturated)

Extractions in “phenol” were extracted using phenol:chloroform:isoamyl alcohol (25:24:1, Tris-saturated, pH 8.0)

^c^ Precipitation reagent was either RT 2-Prop (room temperature isopropanol) or Cold ETOH (ethanol). See Additional file 4: Figure S1

^d^ Precipitation salt was either 3 M sodium acetate (pH 5.2) or 5 M NaCl

#### Method comparison

**Fig. 1:**
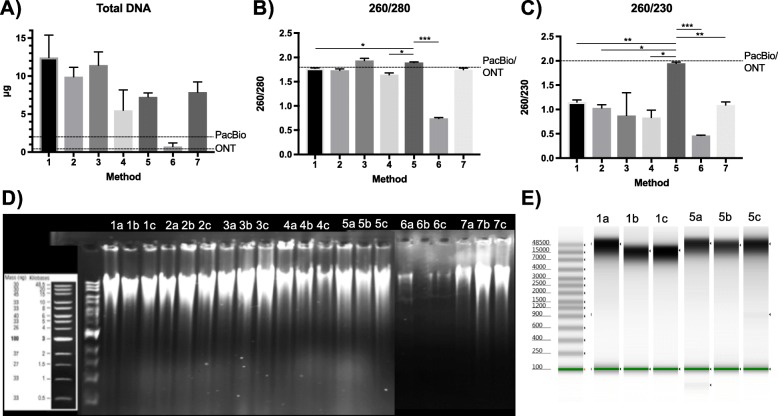
Quantity and quality of DNA by variable methods. **a** Total DNA by Qubit® and **b** 260/280 and **c** 260/230 by NanoDrop 2000 UV-Vis Spectrophotometer. All methods performed in triplicate with error bars (standard deviation). All methods produced sufficient total DNA except Method 6 (**a**). For (**b**) and (**c**), horizontal bars representing significance values for one-way ANOVA with Tukey’s post-hoc multiple comparisons test against Method 5. Significance in *p*-values as follows: 0.033 (*), 0.002 (**), < 0.001 (***). DNA molecular weight demonstrated by (**d**) gel electrophoresis of genomic DNA on a 0.6% ethidium bromide gel of extractions by Methods 1–7 in triplicate (a, b, c) and (**e**) Bioanalyzer of Method 1 and Method 5 in triplicate

Bacterial pellets averaged a normalized “washed weight” of 26.4 mg. With the exception of Method 6, all methods produced sufficient total DNA quantity (Fig. 1A). Method 1 produced the highest total amounts of DNA (mean 12.45 μg, standard deviation [SD] 2.928). All methods with the exception of Method 6 gave sufficient 260/280, indicating low protein contamination overall (Fig. 1B). Method 3 and 5 produced the highest 260/280 measurements, which were significantly higher than other methods (Fig. 1B). Only Method 5 produced sufficient 260/230 for use with long-read sequencers, which was significantly higher than all other methods (Fig. 1C). Despite variations in quantity, all methods produced high MW DNA as evidenced on an agarose gel, indicating preservation of high MW fragments of genomic DNA (Fig. 1D). For deeper comparison, Method 1 and Method 5 DNA extractions were also analyzed on a bioanalyzer (Fig. 1E) with peak means of 29,369 base pairs (SD 20,002 bp) and 51,598 base pairs (SD 5,882 bp), respectively. While there was a trend toward higher MW DNA fragments achieved by Method 5, the differences were not significant (paired t-test, *p* = 0.1450). In short, Method 5 was the only method to produce sufficiently pure DNA for ONT MinION sequencing without requiring additional clean-up steps in all quality and quantity measurements with mean total DNA of 7.263 μg (SD 0.50), mean 260/280 of 1.893 (SD 0.012), and mean 260/230 of 1.947 (SD 0.025).

#### Method validation

**Fig. 2:**
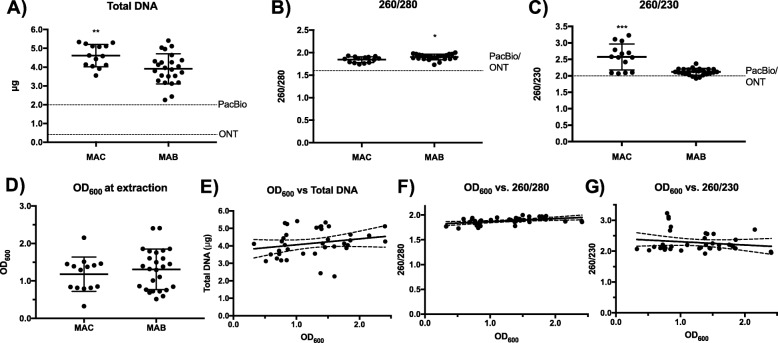
Quantity and quality of DNA extractions of 38 MAC and MABSC clinical isolates using optimized DNA extraction method. **a** Total DNA by Qubit® and **b** 260/280 and **c** 260/230 by NanoDrop 2000 UV-Vis Spectrophotometer. Significance by unpaired t-test with *p*-values as 0.033 (*), 0.002 (**), < 0.001 (***). **d** OD_600_ at the time of extraction by species complex (ns). Scatter plots with lines of best fit for (**e**) OD_600_ versus total DNA (ns), (F) OD_600_ versus 260/280 showing a positive relationship (*p* = 0.0033, *R2* = 0.2051), and OD_600_ versus 260/230

Of 38 clinical isolates from 8 patients extracted with our optimized method (Method 5), 12 isolates were identified as *Mycobacterium avium* complex (MAC, slow-growing), and 26 isolates were *Mycobacterium abscessus* complex (MABSC, rapid-growing) by taxonomic classification. Notably, all 38 extractions using Method 5 yielded sufficient quality and quantity measurements for long-read sequencing without requiring any additional clean-up steps (Fig. 2a–c). All DNA extracts achieved high enough quantity of DNA (mean 4.17 μg, SD 0.80) and quality of DNA with mean 260/230 of 2.29 (SD 0.33) and mean 260/280 of 1.88 (SD 0.069), meeting the required specifications by the ONT manufacturer. Although all DNA extractions were adequate for ONT sequencing, we also found that MABSC samples had significantly higher 260/280 (*p* = 0.01) and total DNA (*p* = 0.007), while MAC samples had significantly higher 260/230 (*p* <  0.001) (Fig. 2a–c). Due to wide variation in starting OD_600_ among NTMs ([0.330–2.409]; mean = 1.225, median = 1.294), we also investigated if OD_600_ correlated with extraction outcomes among all MAC and MABSC samples. While OD_600_ between MAC and MABSC samples did not vary significantly at the time of extraction (Fig. 2d), we observed a positive relationship between OD_600_ and 260/280 for all samples (*p* = 0.0033, *R*^*2*^ = 0.2051) (Fig. 2f). No significant relationship was found between OD_600_ and total DNA or 260/230 (Fig. 2e, g).

#### ONT whole genome sequencing and assembly

Three isolates from MAC single colonies (CHOP101034, CHOP101115, and CHOP101174) were chosen for long-read sequencing as biological replicates. As Method 5 was the only method to produce sufficient quantity and quality of DNA for long-read sequencing, use of an alternative method for comparison required additional clean-up steps (see Additional file 1 for isopropanol clean-up steps). As clean-up of DNA inevitably results in significant loses of total DNA, we chose Method 1 for comparison, as it yielded the highest total DNA. All DNA extracts achieved high enough quantity and quality prior to ONT sequencing. Of note, the ONT MinION sequencer is very sensitive to poor-quality DNA, and samples with low 260/230 will cause osmotic imbalances in the flow cell (Experiment protocol, SQK-RBK-004, ONT).

Despite having sufficient quantity and quality of DNA and using the same library preparation and parameters for both run preparations, the Method 5 sequencing run gave superior total reads and total bases sequenced with significantly higher mean (*p* = 0.0168) and median (*p* = 0.0101) read lengths per barcode (Table 2). To assess for variability between MAC and MAB sequencing, we additionally completed WGS on 8 isolates from 8 different patients selected from the 38 clinical samples mentioned previously. Extraction and sequencing data for this 8-sample run can be found in Additional file 3: Table S2. There were no significant differences between MAC and MAB sequencing results with regard to total reads, total bases, mean or median read lengths, or longest read sequenced (unpaired t-tests). Plots of sequencing run outputs for all ONT MinION runs are displayed in Additional file 4: Figure S1. A complete list of all strains extracted by the optimized extraction method, Method 5, is also available (Additional file 5: Table S4).

**Table 2:** ONT Sequencing Run Statistics

	Total reads	Total bases	Mean read length (bp)	Median read length (bp)	Longest read	Phred score	%Error Probability^a^
Method 1	1,455,977	1,634,706,997	1122.8	751.0	34,548	13	5.011%
Method 5	1,500,966	2,778,573,523	1851.2^*^	1196.0^*^	64,327	14	3.981%

Raw values are listed for each MinION ONT runs using DNA extracted by Method 1 (after additional clean-up) and by Method 5. For statistical analyses comparing Method 1 (*n* = 3) versus Method 5 (*n* = 3), paired-t test comparisons (total reads, total bases, mean read length, median read length, longest read) were completed on basecalled, demultiplexed, and trimmed reads. Mean read length and median read length were significantly longer from Method 5, *p* = 0.0168 and *p* = 0.0101, respectively. Notably, Phred score was higher and error probability lower in the Method 5 run, which may reflect higher quality substrates

^*^ Indicates *p*-value of *p* <  0.05

^a^ Error probability percentage is a function of Mean Phred score, where probability P% = 100^*^10^(−Phred/10)

### Complete genomes

The final, long-read based MAC genome assemblies were complete or near-complete with mean genome size 5.316 Mb and 69.01% GC content (Table 3). Finished genome assemblies compared between two investigated methods of DNA extraction (Method 1 with isopropanol clean-up versus method 5 without clean-up) did not vary significantly by statistical analyses (paired t-tests) with regard to length, contig number, N50, %GC, or coverage (Table 3). As the same short-reads were used for polishing both Method 1 and Method 5 assemblies, direct comparisons were able to be completed without variability introduced by short-read data (Additional file 6: Table S3). All three Method 5 assemblies were complete and circularized, while only 1/3 of Method 1 bacterial chromosomes were complete and circularized. While not reaching statistical significance, Method 5 genomes had lower overall contamination scores with means of 0.9 (Method 5, SD 0) versus 3.3 (Method 1, SD 2.12), *p* = 0.1210. Method 5 genomes had significantly higher fine consistency with a mean of 97.37 (Method 5, SD 0.116) versus 96.2 (Method 1, SD 0.608), *p* = 0.0310.

**Table 3:** Assembly Statistics

	Sample	Length (bp)	Contigs	N50 (Mbp)	%GC	Coverage	Circularized, complete chromosome	Plasmids	Completeness/Contamination^b^	Consistency, Coarse/Fine^c^
Method 1	CHOP101034	5,440,506	8	4.46	68.9	102.3x	No	2	100/2.5	98.7/96.5
	CHOP101115	5,391,592	7	4.04	69.0	85.2x	No	2	100/5.7	98.7/96.6
	CHOP101174	5,171,418	3	5.11	69.1	170.3x	Yes^a^	2	100/1.7	98.2/95.5
Method 5	CHOP101034	5,368,111	3	5.30	69.9	83.1x	Yes^a^	1	100/0.9	98.5/97.3^*^
	CHOP101115	5,393,712	5	5.25	68.9	97.3x	Yes	2	100/0.9	98.8/97.5^*^
	CHOP101174	5,130,681	3	5.10	69.2	75.7x	Yes^a^	2	100/0.9	98.7/97.3^*^

Comparison of genomes assembled from reads generated from Method 1 versus Method 5 sequencing runs showing generally more complete assemblies from Method 5, with all Method 5 genomes producing complete and circularized bacterial chromosomes, while only 1/3 bacterial chromosomes by Method 1 being circular and complete. All listed parameters were evaluated for statistical significance between the two methods. Method 5 genomes had significantly higher fine consistency scores than Method 1 genomes by unpaired t-test, *p* = 0.0310

^*^Indicates significance with *p* < 0.05

^a^Complete and circularized chromosome and plasmids without extra unintegrated plasmid contigs

^b^Completeness is the percentage of genes with universal roles represented in the genome; Contamination approximates the percentage of the genome that is contaminated and is estimated by universal roles that are represented more than once in the genome [37].

^c^Genome consistency estimates the percentage of universal roles expected to be present vs. absent (Coarse) in the genome and universal roles that are present in the exact number (Fine) as expected in the genome [37]

The cost for completing each genome was approximately $280 US dollars. This could be reduced to as low as $80 per genome by including more barcodes in the sequencing run; however, in our experience, increasing barcodes decreases coverage per genome (Additional file 3: Table S2). In summary, our optimized protocol for long-read DNA extraction and our assembly pipeline has allowed us to produce DNA sufficient for long-read sequencing after a single extraction without additional clean-up steps, and furthermore, allows us to present the first publicly-available *M. avium* subspecies *hominissuis* genome assemblies constructed utilizing ONT long-reads.

**Fig. 3:**
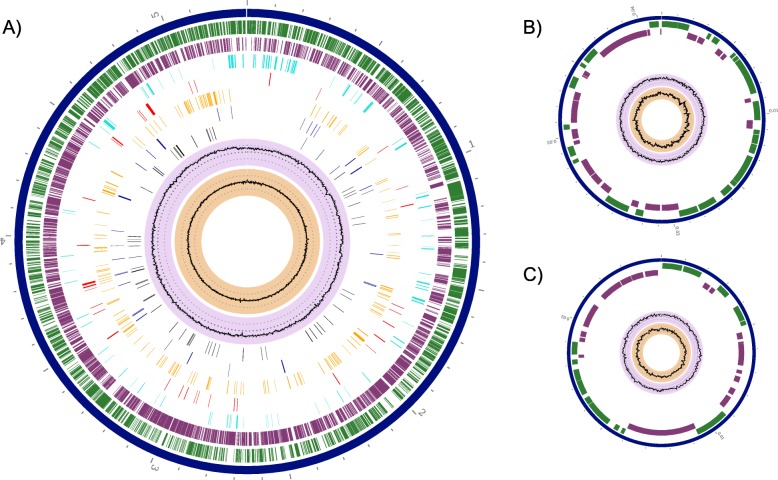
Representative complete and circularized assembly. CHOP101034 bacterial 1 chromosome (**a**), pMARIA (**b**), and pFLAC0181_CHOP101 (**c**). Genome graphics by PATRIC [37], from outside in, position label (Mbp, grey), contigs (dark blue), CDS forward (green), CDS reverse (purple), non-CDS features (teal), antimicrobial resistance genes (red), virulence factor genes (orange), transporters (blue), drug targets (black), GC content (lavender), GC skew (peach)

We present three genome assemblies: CHOP101034, CHOP101115, and CHOP101174, which were all isolated from an adolescent with CF and chronic MAC infection between 2016 and 2017 (Fig. 3). All genomes were identified as *M. avium* subspecies *hominissuis* and are considered clonal isolates by core whole genome alignment (data not shown). Genomic DNA was extracted by Method 5 and sequenced, assembled, and annotated as described above. Two plasmids were identified in the assemblies, including a novel plasmid that we designate here as pMARIA (plasmid *Mycobacterium avium* Replicon [class] 1 [type] a), and a plasmid previously described by Caverly et al., pFLAC0181 (GenBank: CP023150.1, BioSample SAMN07528789, unpublished) identified by WGS from an isolate of *M. intracellularae*.

CHOP101034 consists of a complete, circularized chromosome and two circularized plasmids. CHOP101034 has 5,368,111 base pairs with 68.93% GC content, 5,216 coding sequences (CDS), 99 repeat regions, 47 tRNAs, 3 rRNAs, and mean long-read ONT coverage of 83.12x. The novel plasmid identified, pMARIA, is 41,578 base pairs with 64.55% GC content, 56 CDS, and includes a plasmid stability gene (*parA*), transposases (Tn552, Tn554), and the insertion sequence IS6210. The second plasmid has 100% identity and 100% coverage to pFLAC0181. pFLAC0181_CHOP101 is 24,701 base pairs with 65.33% GC content, 31 CDS, and includes the plasmid stability gene *parA*, transmembrane proteins (*mmpL*, *mmpS*), and a metal sensitive transcriptional repressor. CHOP101115 is composed of 5 contigs including a complete and circularized bacterial chromosome, pMARIA_2, pFLAC0181_CHOP101_2, and two linear contigs that identified most closely as partial plasmid sequences by NCBI blastn [38]. CHOP101115 is 5,393,712 base pairs with 68.94% GC content, 5,228 CDS, 97 repeat regions, 47 tRNAs, 3 rRNAs, and ONT coverage of 97.31x. CHOP101174 is comprised of a circularized bacterial chromosome, pMARIA_1, and pFLAC0181_CHOP101_1. CHOP101174 is 5,130,681 base pairs with 69.18% GC content, 4,952 CDS, 76 repeat regions, 46 tRNAs, 3 rRNAs, and ONT coverage of 75.7x. The pMARIA plasmids were found to be highly similar to each other with percent identity ranging 96.0–99.95%, and pFLAC0181 sequences ranged from 94.72–100% identity by NCBI blastn alignments, with variations representing either sequencing error or natural variation and horizontal gene transfer occurring over time during chronic lung infection [38].

### Data accession

The NTM Project at the Children’s Hospital of Philadelphia, NCBI BioProject PRJNA532547, is available at https://www.ncbi.nlm.nih.gov/sra/PRJNA532547. SRA reads are available for CHOP101034 (BioSample SAMN11403486), CHOP101115 (BioSample SAMN11403599), and CHOP101174 (BioSample SAMN11403589). CHOP101034 is represented by a complete, circularized bacterial chromosome (CP040247), pMARIA (CP040245), and pFLAC0181_CHOP101 (CP040246). CHOP101115 is represented by a complete, circularized bacterial chromosome (CP040255), pMARIA_2 (CP040253), p_FLAC0181_CHOP101_2 (CP040254), and two plasmid fragments (CP040251, CP040252). CHOP101174 is represented by a complete, circularized bacterial chromosome (CP040250), pMARIA_1 (CP040249), and pFLAC0181_CHOP101_1 (CP040248).

## Conclusion

Our protocol produced DNA of sufficient quantity and quality for long-read whole genome sequencing with the ONT MinION sequencer. To demonstrate direct comparisons to alternative methods, we completed DNA extraction with 6 variations of methodology with normalized starting bacterial pellet weights. Method 5 demonstrated superiority as the only method to provide appropriate DNA quality in all tested measurements without requiring any clean-up steps. Method 5 was characterized by early bead-beating in high-SDS concentration, gentle phenol-based extraction, and room temperature isopropanol precipitation. While Method 5 was the only method to use NaCl as the precipitation salt, later direct comparisons of NaCl versus NaOAc alone did not demonstrate any superiority of NaCl. Thus, while either salt is appropriate, we recommend NaCl over NaOAc because it does not require pH titration. During development of our protocol we also trialed an alternative buffer, variable concentrations of lysozyme and proteinase K, variable starting weights of bacterial pellets, extraction without bead-beating, and bead-beating with and without SDS, in addition to all the variables described in this manuscript (some of these variables are represented in Additional file 2: Table S1),

In comparison to the widely-used method by Käser et al. [32] that is useful for short-read sequencing, we noted improvements in the purity of DNA (260/230) with modifications of the composition of lysis buffer (Additional file 1), the timing of bead beating (early vs. late), the use of Phase Lock Gel™ tubes, and the use of room temperature isopropanol as opposed to cold 100% ethanol. Others have shown improved DNA purity with isopropanol extractions compared to cold ethanol extractions with less salt carry-over, albeit at the expense of DNA yields [31, 32]. While Method 1 and 3 gave more total DNA, neither reached a suitable 260/230 absorbance. Thus, our method sacrifices total DNA yield to achieve high DNA purity. Our optimized method was also highly reliable, yielding sufficient quality and quantity for ONT sequencing in all 38 MAC and MABSC clinical isolates, regardless of mycobacterial species or starting OD_600._

The trademark of the mycobacterial cell wall is its hardy, heavily lipophilic exterior. In addition, mycobacterial peptidoglycans are characterized by an oxidation modification rendering lysozyme less effective at cleaving the β [1, 4] linkages between N-acetylmuramic acid and N-acetyl-D-glucosamine residues [39]. Thus, it is no surprise that mechanical cell wall disruption is necessary for DNA extraction. We reasoned that *early* mechanical disruption allows the exterior mycolic acid cell wall and peptidoglycan layer to be broken down first, with subsequent enzymatic digestion with lysozyme and proteinase K to digest the remainder of the cell wall and expose its inner contents. In our preliminary trials, early mechanical disruption demonstrated superiority to late mechanical disruption. Although not achieving statistical significance in the head-to-head comparisons presented here, we have consistently noted increased shearing with late mechanical disruption, resulting in homogenously distributed smears of MW DNA on gel electrophoresis. In addition, we found that the early addition of high concentrations of SDS during early beat-beating was also independently superior to bead-beating without SDS (Additional file 2: Table S1). The detergent properties of SDS likely assist with mechanical lysis and may additionally protect exposed DNA from degradation.

The optimized method presented here is able to produce large amounts of very pure, high molecular weight DNA without extra clean-up steps. The avoidance of clean-up steps is essential because repeat precipitation-based and SPRI bead-based clean-up methods consistently result in the loss of large amounts of DNA. Thus, a single method that is able to produce highly pure DNA without clean-up is critical in cases where larger amounts of DNA are desired for long-read sequencing modalities. Additional modifications in DNA preparation that should be considered are size selection and library kit selection. Size selection was intentionally not completed to avoid introduction of variability that could potentially lead to bias in method comparison. However, size selection should be considered prior to library preparation as it may increase sequenced read lengths. Interestingly, we found that use of SPRI bead-based size selection (used in the 8-sample run) did not significantly improve read lengths when using the ONT 1D Rapid Barcoding Kit (SQK-RBK004), by unpaired t-tests (Additional file 3: Table S2). As this particular kit is transposase-based and introduces breaks in DNA during adapter annealing, we suggest that if longer reads are desired, the Ligation Sequencing Kit could be used to achieve even longer reads. In which case, we recommend SPRI-bead size selection prior to library preparation, as the ligation kit does not require fragmentation for adapter annealing (https://nanoporetech.com/products/kits) [40]. However, even using the rapid kit with induced fragmentation of DNA and no size selection, we were able to complete bacterial genomes with our ONT-based assemblies.

For the three genomes presented, we notice slight variability in genome length. The genomes constructed for the exact same isolate by Method 1 versus Method 5 vary in length by about 1%. For example, CHOP101174 varies by 0.8% in length between the two sequencing methods and both methods produced closed genomes, however the measurements of consistency were higher and contamination lower in the Method 5 genome. While these variations may be a result of sequencing error, they may also be due to an inability to resolve repeat regions and join contigs across regions where sequences have lower quality. In the setting of high GC content genomes with large repeat regions, a larger (and more fragmented) genome likely reflects duplications in repeat regions that cannot be resolved. Differences in genome length between the three isolates may also be a result of natural variation over time. While the 3 isolates sequenced are essentially biological replicates and are from the same patient, they were obtained at different time points during a chronic lung infection, and thus, differences may also reflect adaptation, recombination, insertion, or deletion. Taken as a whole, while the genome quality metrics for Method 5 are only slightly improved compared to Method 1, we favor the length results obtained by Method 5 based on its marginally better quality and closure. In addition, the Method 5 extraction technique is simpler and does not require extra clean-up steps.

Long-read sequencing offers a much higher likelihood than short-read sequencing of producing complete (circularized) genomes. Complete genomes can be used to more readily identify critical genomic characteristics such as extrachromosomal elements, mosaicism/recombination tracks, large repeat regions, duplications, and inversions, all of which likely play critical roles in mycobacterial genomic evolution; these elements can introduce signals (e.g., horizontal gene transfer) that confound phylogenetic inference [19, 28, 29, 41, 42]. Therefore, studies aimed at understanding patterns of transmission and genomic evolution that rely heavily on phylogeny gain power and accuracy when they consider complete genome sequences. Completed genomes also provide optimal reference sequences for comparison of clonal relatives because they contain a more complete picture of genome content and organization.

Our optimized extraction protocol and ONT assembly pipeline presented here were both sufficient and efficient for genome closure at a fraction of the cost and time of other approaches. Undoubtedly, long-read assembled genomes are the way of the future, but regardless of new technologies for cheap and high-fidelity DNA sequencing, we remain at the mercy of the cell wall, and we will continue to be faced with the delicate challenge of mining unscathed DNA from a distinctly robust substrate. We expect that our finely-tuned extraction method will prove to be a valuable tool in the mycobacterial genomics field going forward.

## Methods

### Bacterial growth

Clinical isolates of NTM were grown from frozen stocks to Löwenstein–Jensen slants and sub-cultured to Middlebrook 7H11 plates. Single colonies from 7H11 plates were inoculated in Middlebrook 7H9 broth supplemented with 10% Oleic Albumin Dextrose Catalase (OADC) and incubated at 37 °C shaking for up to 2 weeks (OD_600_ > 0.700). Bacterial cultures were pelleted and stored at − 20 °C until the time of extraction. Initial comparative analyses of methods were done on MAC isolates, while the method validation was completed on both MAC and MABSC isolates.

### DNA extraction method optimization and validation

The following extraction protocol described is our optimized method, “Method 5.” Alterations in Method 5 for comparison are described in Table 1. Method 3 is similar to a standard protocol as described by Käser et al., with the only difference being the composition of the lysis buffer [32]. The comprehensive protocol of Method 5 (optimized method) with thorough descriptions of each step and reagent recipes is provided in Additional file 1.

#### Sample preparation

Bacterial pellets were resuspended and washed in 350 μL of 1X phosphate-buffered saline (PBS) using 2 mL microcentrifuge tubes. Due to variability in starting weights between bacterial isolate cultures, and for the purposes of comparing extraction methods, all weights were normalized after a second PBS wash and “washed weights” were recorded. The samples were heat-inactivated for 60 min at 95 °C, pelleted, and supernatant discarded.

#### “Early” mechanical disruption in SDS followed by enzymatic digestion

Bacterial pellets were resuspended in 400 μL of lysis buffer and 100 μL of 20% SDS. Samples were homogenized with glass beads (four 30-s cycles, maximum setting, Fisher Scientific vortex mixer, MoBio adapter) (150 mg glass beads, 0.1-mm diameter, Research Products International). Subsequently, all vortexing was avoided. Cell walls were additionally lysed in lysozyme (final concentration 10 mg/mL) for 1 h at 37 °C. Proteinase K (final concentration 200 μg/mL) was added and samples were incubated at 37 °C for 90 min with mixing by turning end-over-end by hand to create a homogenous suspension every 30 min. The lysates were centrifuged (2,000 rcf for 10 min followed by 18,000 rcf for 2 min) and supernatants transferred to 2 mL 5Prime Light Phase Lock Gel™ (PLG, QuantaBio) microcentrifuge tubes. Variables tested included enzymatic digestion prior to mechanical disruption (Methods 2, 3) and the amount of matrix material (Method 4).

#### Phenol:chloroform:isoamyl alcohol extraction

To extract DNA, 500 μL of phenol:chloroform:isoamyl alcohol (25:24:1, Tris-saturated, pH 8.0) was added to the PLG tubes. The tubes were rotated on a HulaMixer (ThermoFisher Scientific, United States) at 20 rpm for 20 min and then centrifuged (2,000 rcf for 10 min). The DNA-containing aqueous layer was transferred to a new 2 mL microcentrifuge tube with care to not aspirate the gel layer. Chloroform:isoamyl alcohol (24:1, Tris-saturated) without phenol was tested as a variable (Methods 1, 2, 4, 6, 7).

#### Isopropanol precipitation

For DNA precipitation, 1/10 volume of 5 M sodium chloride (~ 20–45 μL) and 1 volume of room temperature isopropanol (~ 200–450 μL) was added to the samples. DNA was allowed to precipitate overnight at room temperature. The samples were then centrifuged (18,000 rcf for 30 min at 22 °C, to avoid heating), washed with 700 μL 70% ethanol (18,000 rcf for 10 min at 22 °C), and the supernatant carefully discarded, with repeat of washing steps 3 times. The samples were air-dried at room temperature with lids open for 15 min, resuspended in 100 μL of Tris-Cl elution buffer (Qiagen, Germany), eluted overnight at room temperature on a nutator (24 rpm fixed speed, Fisherbrand™); nutator use is optional and only theoretically assists elution. DNA was stored at 4 °C. Variables tested during precipitation include use of cold 100% ethanol (Methods 3, 6) and use of an alternative salt (Methods 1–4, 6, 7).

#### DNA extraction method validation

Clinical samples of MAC (*n* = 12) and MABSC (*n* = 26) were obtained from the clinical microbiology lab at the Children’s Hospital of Philadelphia as frozen stocks collected from clinical isolates between 2011 and 2018. Frozen stocks were streaked to 7H11 plates and incubated at 37 °C for approximately 2–6 weeks. Single colonies were grown in 4 mL 7H9 plus 10% OADC shaking at 37 °C to OD_600_ > 0.500, pelleted, and pellets frozen at 20 °C until the time of extraction. DNA was extracted using Method 5 as described above.

#### Quality measures

DNA was heated to 55–65 °C prior to quality assessment to ensure homogeneity of DNA per quality measurement guidelines [43]. DNA purity was assessed with NanoDrop 2000 UV-Vis Spectrophotometer (ThermoFisher Scientific, United States) by measurements of 260/280 to detect protein contamination and 260/230 to detect contamination by solvents and salts. DNA concentrations were measured with Qubit® 2.0 Fluorometer dsDNA BR Assay (ThermoFisher Scientific, United States). Gel electrophoresis (0.6% agarose ethidium bromide gel, 40 V for 1.5–2 h) estimated MWs and shearing, which was confirmed by 4200 TapeStation system bioanalyzer (Agilient, United States) in selected samples.

### ONT whole genome sequencing and genome construction

#### DNA preparation and sequencing

Three MAC clinical isolates (CHOP101034, CHOP101115, and CHOP101174) were grown as described above and extracted each by Method 1 and by Method 5. Method 1 was chosen for comparative analyses to Method 5 as it had the highest total amount of DNA. Due to inadequate 260/230 of samples extracted by Method 1, these extracts required “clean-up”, which we completed by re-eluting in 100 μL of elution buffer and repeating isopropanol precipitation 2–3 times prior to achieving adequate quality measurements for use in the ONT MinION. DNA from Method 1 (*n* = 3) and Method 5 (*n* = 3) was prepared for WGS with the Rapid Barcoding Kit (SQK-RBK-004, version: RBK_9054_v2_revD_23Jan2018) with starting DNA with 400 ng per sample barcode, 260/280 of ~ 1.8, and 260/230 of 2.0–2.2 (with optional Solid Phase Reversible Immobilization bead step excluded due to barcoding of under 4 samples), and libraries sequenced on the ONT FLO-MIN107 (R9.4) flow cell. DNA from each of the 3 isolates was additionally sequenced by Illumina HiSeq 2500 using the Nextera XT library preparation kit (Illumina, US). Illumina short reads were demultiplexed with DNAbc and trimmed with Trim Galore! with default settings [44, 45].

#### Genome completion

Raw ONT fast5 output was basecalled with Guppy (ONT, v2.3.1 + 1b9405b), trimmed and demultiplexed with Porechop (−b, barcoding mode on, v0.2.4) [46], and fastq reads filtered with Filtlong (--keep_percent 90 --min_length 1000 --target_bases 500,00,000 --trim --split 500, without an external quality reference, v0.2.0) [47]. Genome assemblies were constructed using the tool Unicycler (default settings, --mode normal, v0.4.8-beta) [48], which was created specifically for utilization of ONT long-reads in the assembly of bacterial genomes. With input of basecalled, filtered fastq long-reads, Unicycler constructs a miniasm [49] assembly graph, polishes with Racon [37], and produces assembly graphs that can be viewed in Bandage [48, 50]. We additionally circularized with Circlator (circlator all, v1.5.5) [51] using input of the Unicycler-assembled genome and ONT long-reads corrected by Canu (canu -correct, genomeSize = 5.2 m, errorRate = 0.144, −nanopore-raw, v1.8) [52]. Post-Circlator assemblies were polished with Illumina short-reads using Pilon (--genome --frags, v.1.22) to improve genome quality [53]. Final assemblies were annotated in PATRIC (v3.5.34, patricbrc.org/) [54]. Assemblies were identified by the Kraken2 taxonomic sequence classification system (kraken2 --db bacteria, default settings, v2.0.7-beta) [55]. Coverage was calculated as an average of basecalled and trimmed fastq reads to the corresponding genome assembly using minimap2 alignment and taking the average read coverage of the samtools depth output (minimap2 -ax map-ont; samtools sort; samtools index; samtools depth) [49, 56].

### Statistical analyses

Statistical analyses were completed in Prism 7.0d for Mac OS X (GraphPad Software, La Jolla California USA, www.graphpad.com). Quality and quantity measurements of DNA extractions were compared by one-way ANOVA with post-hoc Tukey’s multiple comparison tests and unpaired t-tests when appropriate. Linear regression line-of-best-fit analyses were completed to investigate the contribution of starting OD_600_ on extraction outcome measures. ONT read qualities were assessed with NanoPack tools [57], assemblies received quality assessments in QUAST (v.5.0.2, quast.bioinf.spbau.ru) [58], and quality measurements for both reads and assemblies were compared with paired and unpaired t-tests when appropriate. Quality measurements of the genomes were additionally conducted by analyzing presence/absence and frequency of universal genes represented in the genomes in PATRIC, which were compared by unpaired t-tests [54].

## Supplementary information

**Additional file 1.** Protocol for DNA Extraction of Nontuberculous Mycobacteria for Long-Read Whole Genome Sequencing.

**Additional file 2: Table S1.** Previous grid experiments. Here we provide an outline of previous extractions, variations in protocols, and extraction quantity and quality measurements that led to the development of our optimized method.

**Additional file 3: Table S2.** DNA extraction and ONT sequencing statistics for additional 8 samples. Eight NTM isolates from 8 different patients were extracted by our optimized method and sequenced on the ONT MinION. Notably, SPRI bead size selection was completed on all samples and input DNA was increased to 1000 μg for this sequencing run. The ONT Rapid Barcoding Kit was again used. There were no significant differences between MAC and MAB strains when comparing any of the listed statistics (unpaired, parametric t-test), nor were there any significant differences in any listed statistics when compared to the previous ONT sequencing run without size selection. Two outliers were observed with higher total reads and total bases (CHOP118112 and CHOP1500921), which may reflect variability of adapter annealing during barcoding preparation. Decreased coverage per barcode is observed compared to the ONT MinION runs with fewer barcoded samples, as expected.

**Additional file 4: Figure S1.** Sequencing statistics produced by NanoPack Tools [56] demonstrating violin plots of (A) read lengths over time, (B) quality over time, (C) log lengths of reads by barcode, and dot plots of (D) read lengths versus average read quality for each ONT MinION run.

**Additional file 5: Table S4.** Strain List of extractions by optimized method (Method 5). Listed below are the source, date collected, and extraction quality measures for all isolates described that were extracted by Method 5.

**Additional file 6: Table S3.** Short-read Statistics. Short-reads were sequenced by Illumina HiSeq 2500 after Nextera XT library preparation, demultiplexed with DNAbc, and trimmed with Trim Galore! [43, 44]. No significant differences were found between the sequenced samples with regard to total reads or quality by Phred score (unpaired t-tests).

## Data Availability

The NTM Project at the Children’s Hospital of Philadelphia, NCBI BioProject PRJNA532547, is available at https://www.ncbi.nlm.nih.gov/sra/PRJNA532547. Please see “Data Accession” in the Results section for identification of reads and genomes associated with specific NCBI BioSamples.

## Abbreviations

CDS: Coding sequences
CF: Cystic fibrosis
COPD: Chronic obstructive pulmonary disease
MABSC: *Mycobacterium abscessus* complex
MAC: *Mycobacterium avium* complex
NTM: Nontuberculous mycobacteria
ONT: Oxford Nanopore Technologies
PacBIO: Pacific Biosciences
SDS: Sodium dodecyl sulfate
SEM: Standard error of the mean
SMRT: Single Molecule Real Time
WGS: Whole genome sequencing
