# Supplementary material for: Complete nontuberculous mycobacteria whole genomes using an optimized DNA extraction protocol for long-read sequencing
Source: BMC Genomics. 2019 Oct 30;20:793. doi: 10.1186/s12864-019-6134-y (PMC6822416; doi:10.1186/s12864-019-6134-y)
Supplement: Supplementary file 1 — Additional file 1. Protocol for DNA Extraction of Nontuberculous Mycobacteria for Long-Read Whole Genome Sequencing. [file 12864_2019_6134_MOESM1_ESM.pdf]

Supplemental Material 1. Protocol for DNA Extraction of Nontuberculous Mycobacteria for Long-Read Whole Genome Sequencing

Bouso, JM and Planet, PJ.

Reagents and Supplies

All reagents should be made with DNase-free water ONLY and always filtered if possible. Lysozyme, SDS, and 70% ETOH preparations should be made on the day of experiment. Lysis buffer may be made prior to day of experiment but RNase A must be added fresh.

Total Lysis Buffer (TLB): 100 mM NaCl, 10 mM Tris-Cl (pH 7.5), 25 mM EDTA (pH 8.0), 0.5% (w/v) Sodium Dodecyl Sulfate, 20 µg/ml Qiagen RNase A (added fresh; filter buffer prior to adding RNase) *Note: This buffer recipe was obtained from protocols.io (36).*
Elution Buffer: Qiagen EB (Tris-Cl)

2 mL microcentrifuge tubes
Phosphate-Buffered Saline
20% Sodium dodecyl sulfate (SDS)
Lysozyme, 100mg/mL working concentration
Proteinase K, 2.5 mg/mL working concentration
25:24:1 Phenol:Chloroform:Isoamyl alcohol (Tris-Saturated, molecular biology grade, buffered to pH 8.0)
Light Phase Lock Gel™ Tubes (PLG, QuantaBio) microcentrifuge tubes
5 M NaCl (can also use 3 M NaOAc, pH 5.2)
Isopropanol (molecular biology grade)
70% EtOH

Preparation of Cell Suspensions from cultured mycobacteria

1. Start with NTM isolates grown in 7H9+ADC (or OADC) media at 37˚C to an OD of 0.500 – 1.200 at 600nm.
   a. From 4-30 mL liquid culture, vortex to homogenous suspension.
      i. At this point you can pipette off necessary amounts for stock for frozen stocks.
      ii. Pellets may also be frozen for future extractions. Store at -20˚C.
   b. Centrifuge tubes at 4500 rpm x 10 minutes and discard supernatant. *Note: Make sure supernatant is not cloudy, sometimes a longer centrifuge is required.*
   c. Weigh pellet. Starting mass should be 20-100 mg wet weight (optimal extractions at ~60-80 mg for wet weight). If you wash in PBS and weigh in a microcentrifuge tube, optimal weights may be 20-30 mg for “washed weight”.
      i. Larger pellets may be divided and extractions performed in parallel; extracted DNA *only from the same single colonies* may be combined later.
      ii. Resuspend pellet in 350 µL of 1X PBS in 2 mL microcentrifuge tubes
2. Heat inactivate/kill the resuspended mycobacteria at 95˚C for 60 minutes
3. Centrifuge for 10 minutes at 4500 rpm and discard supernatant to remove residual PBS. *Note: May require an additional 5-10 minutes if the supernatant is cloudy.*
4. Add 400 µL of lysis buffer plus 100 µL of 20% SDS and mix by pipetting.

“Early” Mechanical Disruption

5. Add 150 mg of matrix material (glass 0.1-mm diameter) and homogenize at 3000 rpm for 4 cycles x 30 seconds each (Fisher Scientific vortexer with MoBio microcentrifuge tube holder adapter; alternative homogenizer of Precellys 24 homogenizer 3 × 30 sec, up to 6800 rpm, Kaser 2010, Ref 32). *Note: Watch the tubes carefully to avoid leakage.*

“Late” Cell wall digestion

6. Add 50 µL of lysozyme (100mg/mL stock; final concentration 10 mg/mL), mix by turning end-over-end by hand several times, and incubate at 37˚C x 1 hour. *Note: All subsequent vortexing should be avoided. Note: Glass beads are still in solution; thus, thorough mixing is important.*
7. Add 40 µL Qiagen Proteinase K (2.5 mg/mL; final concentration of 200 ug/ml), mix by rotating end-over-end by hand, and incubate at 37°C for 90 minutes; every 30 minutes, mix by rotating end-over-end several times (after 30 and 60 minutes).
8. Centrifuge at 4500 rpm x 10 minutes followed by 14000 rpm x 2 minutes. *Note: The beads will not pellet to the bottom and so be very careful removing from the centrifuge to not disturb the beads.*
9. Very carefully, without agitating the beads, pipet off supernatant and transfer to PLG tubes.

Chloroform:isoamyl alcohol extraction

10. Immediately prior to use, pellet Phase Lock Gel (PLG) at 12,000 - 16,000 x g for 20 to 30 seconds.
11. Add 500 µL phenol:chloroform:isoamyl alcohol (25:24:1, buffered to pH of 8.0) to the PLG tubes.
12. Place PLG tubes on a HulaMixer at 20 rpm for 10 minutes to thoroughly mix organic and aqueous phases; this should form a homogenous suspension.
13. Centrifuge at 4500 rpm for 10 minutes. The PLG gel will form a barrier between the aqueous and organic phases.
14. Carefully pipet off nucleic acid-containing aqueous upper phase to fresh 2 mL microcentrifuge tubes. *Note: Aqueous layer should be about 200-450 µL.*

Isopropanol precipitation and Elution

15. Add 1/10 volume (20-45 µL) of 5 M NaCl followed by 1 volume (200-450 µL) of isopropanol.
16. Incubate overnight at room temperature.
17. Centrifuge at 14,000 rpm for 30 min at 20-22°C. *Note: Temperature control is to prevent overheating.*
18. Wash with 700 µL of 70% ethanol and centrifuge at 14,000 rpm for 10 minutes. *Note: Keep the tubes positioned in the same direction so that you always know where the pellet "should be" as it is often invisible!*
19. Do an addition 2-3 more washes (700 µL of 70% ETOH, spin x 14000rpm x 10min and discard supernatant). *Note: You may want to save the first supernatant until your extraction is complete to ensure no loss of DNA.*
20. Let the remaining ethanol evaporate by leaving at room temperature for 15 minutes with tube lids cracked. *Note: Some water may remain. Tubes can be dumped upside down onto Kim wipes to help get rid of water if you desire but any ethanol should evaporate off after 15 minutes.*
21. Add 50-100 µl of warmed EB (that has been heated to 65°C x 10 minutes)
22. Leave overnight at room temperature (optional: can allow to rotate on a nutator).
23. Store at 4°C. (Optional: typically best results seen after allowing to continue to elute at 4°C for 3-4 days prior to quality assessment.

Quality assessments

24. Heat DNA to 65°C x 1 hour. Quality assessments should be completed with Nanodrop Spectrophotometry, Qubit® fluorometry, and gel electrophoresis. We recommend 16S-based PCR with Sanger sequencing prior to WGS to ensure no contamination.
    a. Nanodrop:
        i. Goal: 260/280 of 1.6-1.8
        ii. Goal 260/230 of 2.0.
    b. Qubit: high concentrations/high quantity.
        i. PacBio goal: ideal total DNA is >5µg
        ii. MinION goal: >400ng with minimum concentration of >53 ng/µL
    c. Gel: 0.6% agarose gel, 0.1% EtBr, run low and slow (40V x 1.5-2 hours).
        i. Goal: Large bright top-heavy smears/bands on gel that are >48kb on extend ladder.
25. If sample quality is suboptimal, options for clean-up include:
    d. Longer elution at 4°C.
    e. Repeat isopropanol precipitation
        i. Best to have starting volume of 100µL DNA at least. Reaction should be scaled to reaction volume (start at step 15).
    f. SPRI-bead based clean-up
