# Supplementary material for: Complete nontuberculous mycobacteria whole genomes using an optimized DNA extraction protocol for long-read sequencing
Source: BMC Genomics. 2019 Oct 30;20:793. doi: 10.1186/s12864-019-6134-y (PMC6822416; doi:10.1186/s12864-019-6134-y)
Supplement: Supplementary file 2 — Additional file 2: Table S1. Previous grid experiments. Here we provide an outline of previous extractions, variations in protocols, and extraction quantity and quality measurements that led to the development of our optimized method. [file 12864_2019_6134_MOESM2_ESM.pdf]

**Supplemental Table 1. Previous grid experiments.** Here we provide an outline of previous extractions, variations in protocols, and extraction quantity and quality measurements that led to the development of our optimized method.

| CHOP ID | Pellet weight | Lysis buffer[+] | Bead-beating[*] | Bead quantity | SDS | Phenol[†] | Precipitation Temp/ Reagent[‡] | Precipitation salt[‖] | Total DNA | 260/280 | 260/230 | Gel visual peak (kb) |
|---|---|---|---|---|---|---|---|---|---|---|---|---|
| 101011 | n/a | TLB | None | n/a | Late | No Phenol | Cold ETOH | NaOAc | 0.092 | 0.86 | 0.28 | Faint/no peak |
| 101031 | n/a | TLB | None | n/a | Late | No Phenol | Cold ETOH | NaOAc | 0.051 | 1.11 | 0.36 | Faint/no peak |
| 101101 | n/a | TLB | None | n/a | Late | No Phenol | Cold ETOH | NaOAc | 0.059 | 1.62 | 1.06 | Faint/no peak |
| 101111 | n/a | TLB | None | n/a | Late | No Phenol | Cold ETOH | NaOAc | 0.145 | 1.54 | 0.58 | Faint/no peak |
| 101151 | n/a | TLB | None | n/a | Late | No Phenol | Cold ETOH | NaOAc | 0.205 | 1.69 | 1.47 | Faint/no peak |
| 101161 | n/a | TLB | None | n/a | Late | No Phenol | Cold ETOH | NaOAc | 0.206 | 1.53 | 0.67 | Faint/no peak |
| 101171 | n/a | TLB | None | n/a | Late | No Phenol | Cold ETOH | NaOAc | 0.261 | 1.81 | 1.46 | Faint/no peak |
| 101201 | n/a | TLB | None | n/a | Late | No Phenol | Cold ETOH | NaOAc | 0.198 | 1.52 | 0.65 | Faint/no peak |
| 101211 | n/a | TLB | None | n/a | Late | No Phenol | Cold ETOH | NaOAc | 0.130 | 1.62 | 0.56 | Faint/no peak |
| 101241 | n/a | TLB | None | n/a | Late | No Phenol | Cold ETOH | NaOAc | 0.074 | 2.18 | 0.95 | Faint/no peak |
| 101175a | 35 mg | KSB | Early | 150 mg | Early | No Phenol | Cold ETOH | NaOAc | 2.324 | 1.06 | n/a | >48 |
| 101175b | 35 mg | KSB | None | n/a | Early | No Phenol | Cold ETOH | NaOAc | 0.159 | 0.70 | n/a | empty |
| 101175c | 35 mg | TLB | Early | 150 mg | Early | No Phenol | Cold ETOH | NaOAc | 5.908 | 0.83 | n/a | >48 |
| 101175d | 35 mg | TLB | None | n/a | Early | No Phenol | Cold ETOH | NaOAc | 0.410 | 0.67 | n/a | empty |
| 101032a | 30 mg | KSB | None | n/a | Early | No Phenol | Cold ETOH | NaOAc | 0.267 | 0.80 | n/a | empty |
| 101032b | 30 mg | TLB | None | n/a | Early | No Phenol | Cold ETOH | NaOAc | 0.178 | 0.70 | n/a | empty |
| 101115a | 55 mg | TLB | Early | 100 mg | Early | No Phenol | Cold ETOH | NaOAc | 2.240 | 0.89 | 0.5 | >48 |
| 101115b | 55 mg | TLB | Early | 150 mg | Early | No Phenol | Cold ETOH | NaOAc | 7.210 | 2.33 | -5.61 | >48 |
| 101155a | 70 mg | TLB | Early | 100 mg | Early | No Phenol | Cold ETOH | NaOAc | 3.409 | 1.32 | -10.38 | >48 |
| 101155b | 70 mg | TLB | Early | 150 mg | Early | No Phenol | Cold ETOH | NaOAc | 9.660 | 1.02 | 0.61 | >48 |
| 101204a | 70 mg | TLB | Early | 100 mg | Early | No Phenol | Cold ETOH | NaOAc | 4.459 | 2.26 | -0.66 | >48 |
| 101204b | 70 mg | TLB | Early | 150 mg | Early | No Phenol | Cold ETOH | NaOAc | 10.360 | 1.81 | 7.69 | >48 |
| 101245a | 60 mg | TLB | Early | 100 mg | Early | No Phenol | Cold ETOH | NaOAc | 2.219 | 0.73 | 0.38 | >48 |
| 101245b | 60 mg | TLB | Early | 150 mg | Early | No Phenol | Cold ETOH | NaOAc | 7.210 | 0.78 | 0.35 | >48 |
| 101033a | 60 mg | TLB | Early | 150 mg | Early | Phenol | RT/2-Prop | NaCl | 5.115 | 1.78 | 2.79 | >48 |
| 101033b | 154mg | TLB | Early | 150 mg | Early | Phenol | RT/2-Prop | NaCl | 5.115 | 1.74 | 3.06 | >48 |
| 101034a | 100 mg | TLB | Early | 150 mg | Early | Phenol | RT/2-Prop | NaCl | 4.433 | 1.86 | 2.42 | >48 |
| 101034b | 49 mg | TLB | Early | 150 mg | Early | Phenol | RT/2-Prop | NaCl | 4.620 | 1.87 | 2.58 | >48 |
| 101115 | 93 mg | TLB | Early | 150 mg | Early | Phenol | RT/2-Prop | NaCl | 4.587 | 1.90 | 2.56 | >48 |
| 101174a | 31 mg | TLB | Early | 150 mg | Early | Phenol | RT/2-Prop | NaCl | 4.136 | 1.79 | 3.23 | >48 |
| 101174b | 77 mg | TLB | Early | 150 mg | Early | Phenol | RT/2-Prop | NaCl | 4.048 | 1.84 | 2.67 | >48 |
| 101931 | 76 mg | TLB | Early | 150 mg | Early | Phenol | RT/2-Prop | NaCl | 4.114 | 1.87 | 2.70 | >48 |
| 101932 | 103 mg | TLB | Early | 150 mg | Early | Phenol | RT/2-Prop | NaCl | 3.124 | 1.93 | 2.56 | >48 |
| 101941 | 122 mg | TLB | Early | 150 mg | Early | Phenol | RT/2-Prop | NaCl | 3.278 | 1.76 | 3.11 | >48 |
| 1011432 | 105 mg | TLB | Early | 150 mg | Early | Phenol | RT/2-Prop | NaCl | 3.168 | 1.77 | 2.07 | >48 |

[+] Total Lysis Buffer (TLB), developed by Josh Quick and available on protocols.io, is described in our full protocol (Supplemental Material 1) (36). KSB refers to a "Käser specific buffer", which is described in Käser et al. (32).

[*] "Early" bead-beating refers to the timing prior *to* enzymatic digestion; "Late" bead-beating refers to timing after enzymatic digestion. All Early bead-beating was done in high SDS concentration.

† DNA extractions in “no phenol” were extracted as described in Methods with chloroform:isoamyl alcohol (24:1, Tris-saturated). Extractions in “phenol” were extracted using phenol:chloroform:isoamyl alcohol (25:24:1, Tris-saturated, pH 8.0)

‡ Precipitation reagent was either RT 2-Prop (room temperature isopropanol) or Cold ETOH (ethanol).

‖ Precipitation salt was either 3 M sodium acetate (pH 5.2) or 5 M NaCl.
