# Supplementary material for: Complete nontuberculous mycobacteria whole genomes using an optimized DNA extraction protocol for long-read sequencing
Source: BMC Genomics. 2019 Oct 30;20:793. doi: 10.1186/s12864-019-6134-y (PMC6822416; doi:10.1186/s12864-019-6134-y)
Supplement: Supplementary file 3 — Additional file 3: Table S2. DNA extraction and ONT sequencing statistics for additional 8 samples. Eight NTM isolates from 8 different patients were extracted by our optimized method and sequenced on the ONT MinION. Notably, SPRI bead size selection was completed on all samples and input DNA was increased to 1000 μg for this sequencing run. The ONT Rapid Barcoding Kit was again used. There were no significant differences between MAC and MAB strains when comparing any of the listed statistics (unpaired, parametric t-test), nor were there any significant differences in any listed statistics when compared to the previous ONT sequencing run without size selection. Two outliers were observed with higher total reads and total bases (CHOP118112 and CHOP1500921), which may reflect variability of adapter annealing during barcoding preparation. Decreased coverage per barcode is observed compared to the ONT MinION runs with fewer barcoded samples, as expected. [file 12864_2019_6134_MOESM3_ESM.pdf]

Supplemental Table 2. DNA extraction and ONT sequencing statistics for additional 8 samples. Eight NTM isolates from 8 different patients were extracted by our optimized method and sequenced on the ONT MinION. Notably, SPRI bead size selection was completed on all samples and input DNA was increased to 1000 µg for this sequencing run. The ONT Rapid Barcoding Kit was again used. There were no significant differences between MAC and MAB strains when comparing any of the listed statistics (unpaired, parametric t-test), nor were there any significant differences in any listed statistics when compared to the previous ONT sequencing run without size selection. Two outliers were observed with higher total reads and total bases (CHOP118112 and CHOP1500921), which may reflect variability of adapter annealing during barcoding preparation. Decreased throughput is observed compared to the ONT MinION runs with fewer barcoded samples, as expected.

	NTM species complex	Total DNA (µg)	260/280	260/230	Total reads	Total bases	Mean read length (bp)	Median read length (bp)	Longest read	Phred score	%Error Probability‡
CHOP101931	MAC	8.88	1.8	2.04	64,951	123182184	1,896.50	1201	44026	13	5.011%
CHOP1121241	MAC	4.47	1.91	2.08	155515	230877806	1,484.60	886	33612	13	5.011%
CHOP1131461	MAB	5.85	1.91	2.06	96795	134789658	1,392.50	847	30730	13	5.011%
CHOP1181112	MAB	11.22	1.91	2.13	367144	499130372	1,359.50	873	29997	13	5.011%
CHOP1221001	MAB	7.44	1.91	2.12	114012	117440530	1,030.10	637	18290	13	5.011%
CHOP1301681	MAC	12.48	1.93	2.09	113689	221631613	1,657.80	1023	40205	13	5.011%
CHOP1500921	MAC	6.06	1.92	2.1	229903	588298373	2,558.90	1318	59632	13	5.011%
CHOP1511191	MAC	2.814	1.89	2.1	128596	161015783	1,252.10	778	23217	13	5.011%

† Coverage per sequenced genome, calculated by C = LN/G, where L=average read length, N=number of reads, G=genome size of 5.2 Mb.

‡ Error probability percentage is a function of Mean Phred score, where probability P%=100*10^(-Phred/10).
