# Supplementary material for: Complete nontuberculous mycobacteria whole genomes using an optimized DNA extraction protocol for long-read sequencing
Source: BMC Genomics. 2019 Oct 30;20:793. doi: 10.1186/s12864-019-6134-y (PMC6822416; doi:10.1186/s12864-019-6134-y)
Supplement: Supplementary file 4 — Additional file 4: Figure S1. Sequencing statistics produced by NanoPack Tools [56] demonstrating violin plots of (A) read lengths over time, (B) quality over time, (C) log lengths of reads by barcode, and dot plots of (D) read lengths versus average read quality for each ONT MinION run. [file 12864_2019_6134_MOESM4_ESM.pdf]

Supplemental Figure 1. Sequencing statistics produced by NanoPack Tools (56) demonstrating violin plots of (A) read lengths over time, (B) quality over time, (C) log lengths of reads by barcode, and dot plots of (D) read lengths versus average read quality for each ONT MinION run.

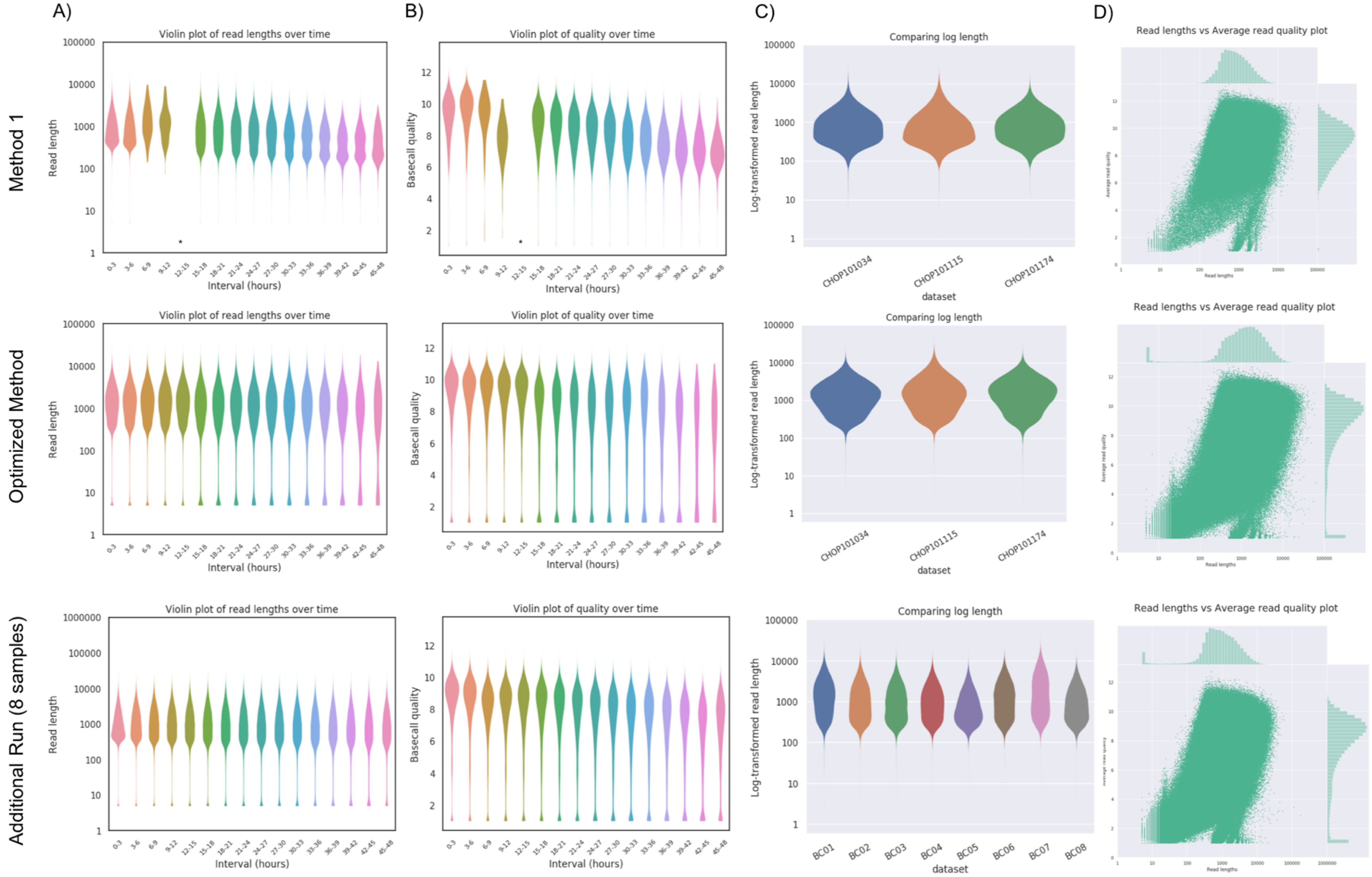

* Indicates a 2-hour period where sequencing stopped due to script error and was restarted manually. This run was extended past the 48-hour run time to increase read output approximately equal to the amount of time of the previous delay.
