# Supplementary material for: Complete nontuberculous mycobacteria whole genomes using an optimized DNA extraction protocol for long-read sequencing
Source: BMC Genomics. 2019 Oct 30;20:793. doi: 10.1186/s12864-019-6134-y (PMC6822416; doi:10.1186/s12864-019-6134-y)
Supplement: Supplementary file 5 — Additional file 5: Table S4. Strain List of extractions by optimized method (Method 5). Listed below are the source, date collected, and extraction quality measures for all isolates described that were extracted by Method 5. [file 12864_2019_6134_MOESM5_ESM.pdf]

Supplemental Table 4. Strain List of extractions by optimized method (Method 5). Listed below are the source, date collected, and extraction quality measures for all isolates described that were extracted by Method 5.

Sample	NTM species complex	Source	Primary diagnosis	Date Collected	Total DNA (µg) (Qubit)	260/280 (Nanodrop)	260/230 (Nanodrop)
CHOP101034	MAC	Human	Cystic Fibrosis	2017-02-13	5.247	1.86	2.42
CHOP101115	MAC	Human	Cystic Fibrosis	2017-08-01	5.302	1.90	2.56
CHOP101174	MAC	Human	Cystic Fibrosis	2016-08-24	5.082	1.84	2.67
CHOP101033a	MAC	Human	Cystic Fibrosis	2017-02-13	5.115	1.78	2.79
CHOP101033b	MAC	Human	Cystic Fibrosis	2017-02-13	5.115	1.74	3.06
CHOP101034a	MAC	Human	Cystic Fibrosis	2017-02-13	4.433	1.86	2.42
CHOP101034b	MAC	Human	Cystic Fibrosis	2017-02-13	4.620	1.87	2.58
CHOP101115	MAC	Human	Cystic Fibrosis	2017-08-01	4.587	1.90	2.56
CHOP101174a	MAC	Human	Cystic Fibrosis	2016-08-24	4.136	1.79	3.23
CHOP101174b	MAC	Human	Cystic Fibrosis	2016-08-24	4.048	1.84	2.67
CHOP101931	MAC	Human	Cystic Fibrosis	2013-11-05	4.114	1.87	2.70
CHOP101932	MAC	Human	Cystic Fibrosis	2013-11-05	3.124	1.93	2.56
CHOP101941	MAC	Human	Cystic Fibrosis	2013-11-02	3.278	1.76	3.11
CHOP1011432	MAC	Human	Cystic Fibrosis	2017-06-07	3.168	1.77	2.07
CHOP1081071	MAB	Human	Cystic Fibrosis	2018-08-08	2.431	1.73	2.03
CHOP112972	MAC	Human	Cystic Fibrosis	2014-02-21	2.255	1.86	2.10
CHOP1121241	MAB	Human	Cystic Fibrosis	2014-12-08	5.335	1.84	2.05
CHOP1131081	MAB	Human	Cystic Fibrosis	2015-02-17	4.961	1.78	1.92
CHOP1131151	MAB	Human	Cystic Fibrosis	2014-10-07	5.038	1.86	2.08
CHOP1131461	MAB	Human	Cystic Fibrosis	2017-10-24	5.115	1.89	2.09
CHOP118501	MAB	Human	Cystic Fibrosis	2011-12-13	5.412	1.94	2.20
CHOP118911	MAB	Human	Cystic Fibrosis	2013-05-14	4.246	1.90	2.12
CHOP118951	MAC	Human	Cystic Fibrosis	2018-06-21	4.037	1.88	1.98

CHOP118952	MAB	Human	Cystic Fibrosis	2018-06-21	4.642	1.90	2.02
CHOP1181111	MAB	Human	Cystic Fibrosis	2014-10-08	4.257	1.86	1.95
CHOP1181112	MAB	Human	Cystic Fibrosis	2014-10-08	4.312	1.93	2.15
CHOP1181161	MAB	Human	Cystic Fibrosis	2014-10-10	4.367	1.98	2.37
CHOP122621	MAB	Human	Cystic Fibrosis	2012-06-23	4.125	1.88	2.10
CHOP122841	MAB	Human	Cystic Fibrosis	2018-02-19	3.872	1.91	2.09
CHOP1221001	MAB	Human	Cystic Fibrosis	2013-07-31	3.795	2.00	2.25
CHOP1221061	MAB	Human	Cystic Fibrosis	2013-09-09	3.960	1.97	2.21
CHOP1221381	MAB	Human	Cystic Fibrosis	2014-12-08	3.916	1.97	2.19
CHOP1221671	MAB	Human	Cystic Fibrosis	2013-07-12	3.553	1.89	2.15
CHOP1221821	MAB	Human	Cystic Fibrosis	2013-06-19	3.509	1.97	2.20
CHOP1301681	MAB	Human	Cystic Fibrosis	2013-06-12	3.718	1.92	2.10
CHOP1301861	MAC	Human	Cystic Fibrosis	2013-06-12	3.531	1.91	2.09
CHOP1511091	MAB	Human	Cystic Fibrosis	2015-03-14	3.553	1.92	2.14
CHOP1511191	MAB	Human	Cystic Fibrosis	2014-12-12	3.179	1.92	2.12
CHOP1511331	MAB	Human	Cystic Fibrosis	2015-03-15	3.278	1.94	2.21
CHOP1511391	MAB	Human	Cystic Fibrosis	2015-03-16	3.168	1.95	2.20
CHOP1511631	MAB	Human	Cystic Fibrosis	2014-07-10	2.431	1.87	2.07
