# Supplementary material for: Complete nontuberculous mycobacteria whole genomes using an optimized DNA extraction protocol for long-read sequencing
Source: BMC Genomics. 2019 Oct 30;20:793. doi: 10.1186/s12864-019-6134-y (PMC6822416; doi:10.1186/s12864-019-6134-y)
Supplement: Supplementary file 6 — Additional file 6: Table S3. Short-read Statistics. Short-reads were sequenced by Illumina HiSeq 2500 after Nextera XT library preparation, demultiplexed with DNAbc, and trimmed with Trim Galore! [43, 44]. No significant differences were found between the sequenced samples with regard to total reads or quality by Phred score (unpaired t-tests). [file 12864_2019_6134_MOESM6_ESM.pdf]

Supplemental Table 3. Short-read Statistics. Short-reads were sequenced by Illumina HiSeq 2500 after Nextera XT library preparation, demultiplexed with DNAbc, and trimming with Trim Galore! (42, 43). No significant differences were found between the sequenced samples with regard to total reads or quality by Phred score (unpaired t-tests).

Sample	Avg. read length (bp)	Total reads	% GC	Avg. Phred Score
CHOP101034	126	2421793	67	36.9
CHOP101115	126	2485526	65	36.9
CHOP101174	126	2348600	67	36.9
